# A mouldable fibreglass backslab device as a novel approach to offload chronic plantar foot ulcers: A retrospective observational audit

**DOI:** 10.1002/jfa2.70001

**Published:** 2024-08-21

**Authors:** Melissa Ting, Ivana Ferreira, Jonathan Hiew, Mahalia McEvoy, Gabrielle Tan, Priyal Shah, Eugenie Nicolandis, Emma J. Hamilton, Jens Carsten Ritter, Michael Nicolaou, Laurens Manning

**Affiliations:** ^1^ Multidisciplinary Diabetes Foot Unit Fiona Stanley Hospital Perth Western Australia Australia; ^2^ Department of Podiatry Fiona Stanley Hospital Perth Western Australia Australia; ^3^ Department of Endocrinology and Diabetes Fiona Stanley Hospital Perth Western Australia Australia; ^4^ Medical School The University of Western Australia Perth Western Australia Australia; ^5^ Department of Vascular Surgery Fiona Stanley Hospital Perth Western Australia Australia; ^6^ Faculty of Health Sciences Curtin University Perth Western Australia Australia; ^7^ Department of Orthotics and Prosthetics Fiona Stanley Hospital Perth Western Australia Australia; ^8^ Infectious Diseases Department Fiona Stanley Hospital Perth Western Australia Australia

**Keywords:** diabetic foot, foot ulcer, offloading, retrospective studies, ulcer healing

## Abstract

**Background:**

Pressure offloading is a critical component of plantar foot ulcer management, including diabetes‐related foot ulcers (DFU). Conventional offloading options such as total contact casting and removable knee‐high walkers may be unsuitable or unsuccessful in patients with morbid obesity, intermittent lower limb oedema, high exudative wounds or poor mobility. A mouldable fibreglass backslab device (BSD) may be a practical alternative to be considered in these situations.

**Methods:**

Data were retrospectively collected on 28 patients (29 foot ulcers) with non‐healing ulcers who received a BSD to offload their foot ulcer as an extension to standard offloading care. Baseline data included: patient demographics, type of offloading prior to BSD application, date of ulcer onset, days ulcer present prior to BSD application and ulcer size at BSD initiation. Measures of success included ulcer size reduction 12 weeks post‐BSD application, time to complete ulcer healing in BSD, time to 50% reduction in ulcer size post‐BSD application and total number of days ulcer present.

**Results:**

The median (IQR) ulcer area and ulcer duration at baseline for 19 patients (20 ulcers) who used the BSD was 1.65 (0.4–3.8) cm^2^ and 531 (101–635) days. At 12 weeks, the median (IQR) ulcer area was 0.3 (0–0.55) cm^2^ with a median (IQR) reduction of 97 (80–100) %. Nine (45%) ulcers achieved complete wound healing (100% reduction in wound size) at 12 weeks post‐BSD application, and the remaining 11 (55%) ulcers achieved at least 50% reduction in wound size. The median (IQR) time to complete wound healing and 50% reduction in wound size was 71 (35–134) days and 24 (15–44) days, respectively. Nine patients ceased use of the BSD and reverted to conventional offloading before their wounds had healed. Of these, four patients achieved a 50% reduction in wound size at the 12‐week mark with conventional offloading.

**Conclusion:**

Our preliminary data suggests that a mouldable fibreglass BSD may be a practical offloading option in the management of DFUs, especially when conventional offloading methods are unsuccessful, unsuitable or unacceptable to patients. Higher level evidence is required to demonstrate suitability or efficacy of the BSD compared to current evidence‐based recommended offloading methods.

**Abbreviations**
BSD: backslab device
DFU: diabetes‐related foot ulcer
IPJ: interphalangeal joint
i‐TCC: instant total contact cast
MTP: metatarsophalangeal
SMHS: South Metropolitan Health Service
TCC: total contact cast
WIfI: Wound, Ischemia and foot Infection classification system

## 1 INTRODUCTION

Effective pressure offloading is the cornerstone of management for plantar foot ulcers, including diabetes‐related foot ulcers (DFUs) [1, 2, 3, 4, 5, 6]. Offloading refers to an intervention that relieves, reduces or redistributes high plantar tissue stress and pressures in the foot in order to achieve ulcer healing or prevent foot ulcers in high‐risk patients [1, 2, 3, 6, 7].

Current offloading guidelines recommend the use of a non‐removable knee‐high device, such as a total contact cast (TCC) or i‐TCC (instant TCC), as first choice in offloading plantar DFUs [1, 2, 6, 8]. Where a non‐removable knee‐high device is contraindicated or not tolerated, removable knee‐high or ankle‐high offloading devices are recommended as the next best option [1, 2, 6, 8]. While knee‐high devices have been shown to be more effective at reducing peak plantar pressures and weight‐bearing activity compared to ankle‐high devices, the evidence also suggests that knee‐high devices are less likely to be worn compared to ankle‐high devices, thus reducing its effectiveness [1, 6]. There currently exists a gap in the offloading strategies for patients presenting with morbid obesity, intermittent oedema in the lower limb, or large/wide feet from severe deformity, who are contraindicated for a TCC and are unable to fit into a removable knee‐high device. Offloading in these patients is often limited to ankle‐high devices or custom‐made therapeutic footwear, which have been shown to be less effective in providing the required offloading for wound healing [1, 6]. Non‐weight bearing by means of bedrest is not recommended due to physical deconditioning and its negative impact on bone mineral density [9]. Furthermore, the use of a wheelchair or a knee scooter is often impractical [1, 3] depending on house layout and general terrain.

The backslab device (BSD) consists of a strip of fibreglass that is moulded and secured to the patient's foot and lower leg with a bandage and used in conjunction with a post‐operative shoe. It is a novel concept that has been used as part of standard clinical care at the Fiona Stanley Hospital (FSH) Multidisciplinary Diabetes Foot Unit (MDFU) and Podiatry clinics to offload challenging wounds in patients who were contra‐indicated for TCC, or in patients who did not tolerate the use of TCC or removable knee‐high devices, or in patients who had ‘hard‐to‐heal’ wounds despite the use of conventional offloading and good standard care [10]. We sought to do a retrospective audit of BSD use and outcomes from the period 1 January–31 December 2021 which is presented in this paper, in addition to a practical guide to the application of the BSD. The authors initiated this with the assumption that a mouldable, removable fibreglass BSD could be used as an effective offloading option in addition to current offloading practices [6] in patients with chronic plantar DFUs.

## 2 METHODS

### 2.1 Data collection

This is a retrospective, observational audit of patients attending both the Fiona Stanley Hospital Podiatry and MDFU clinics (South Metropolitan Health Service) who used the BSD as part of standard of care for their plantar neuropathic DFU from 1 January 2021 to 31 December 2021. All data collected were collated and managed on Microsoft Excel. Reportable baseline data included patient demographics, type of offloading prior to BSD application, ulcer location, date of ulcer onset, days ulcer present prior to BSD application and ulcer size at BSD initiation. Measures of success included ulcer size reduction 12 weeks post‐BSD application, time to complete ulcer healing in BSD, time to 50% reduction in ulcer size post‐BSD application, and total number of days ulcer present.

### 2.2 Ethics

The current audit is a sub‐study of the Audit of MDFU Services at the Fiona Stanley and Fremantle Hospitals. Ethical approval was obtained from the South Metropolitan Health Human Research Ethics Committee (RGS0000003204). Written, informed consent for publication of patient images was provided by the patient.

### 2.3 Clinical procedures

**FIGURE 1:**
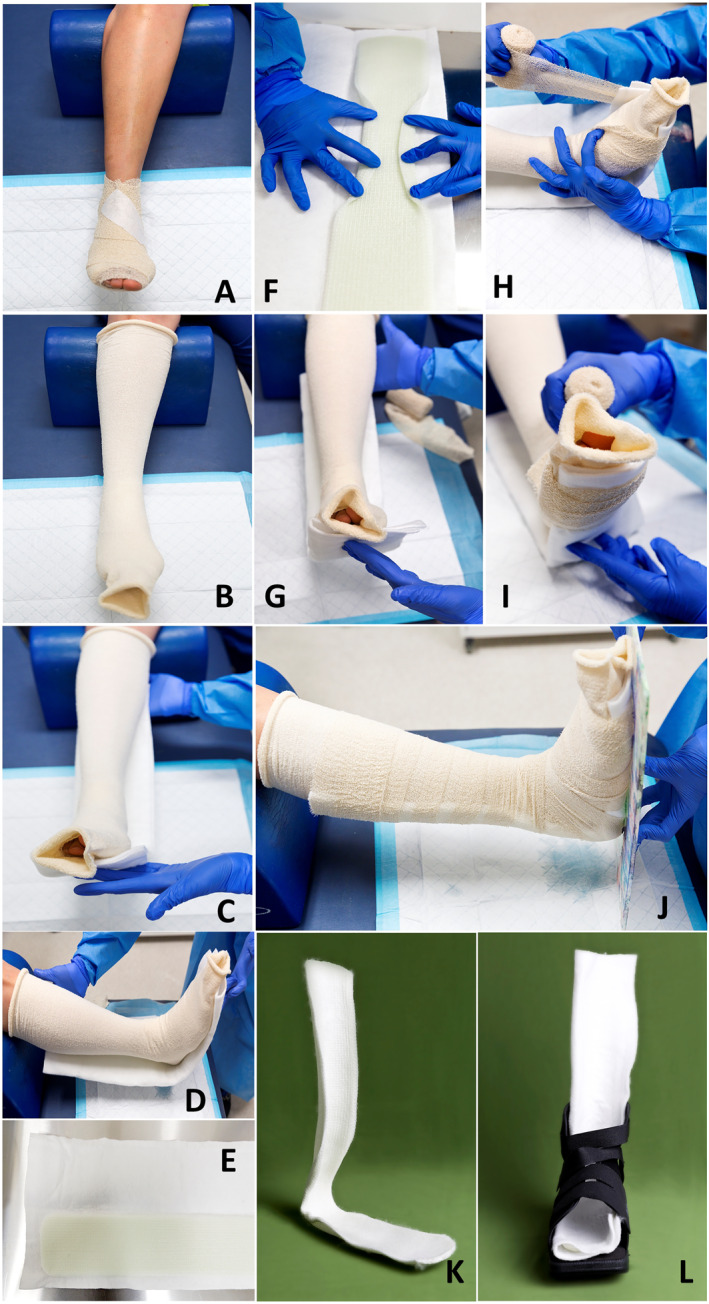
A step‐by‐step guide to apply a moldable fibreglass BSD is pictured above. The foot ulcer should be appropriately dressed prior to BSD application (A). A tubular stockinette is placed over the foot and leg (B). A strip of fibreglass is measured from the plantar forefoot/sulcus to the middle of the calf, taking note of where the heel is located on the splint (C and D). Edges of the splint should be trimmed and rounded off to approximately half an inch within the cotton padding (E). Edges of the splint (where the heel is) are folded inwards, the splint is sprayed with water for activation and the cotton padding replaced (F). This is then placed against the patient's foot and leg, ensuring that any folds are placed away from the skin and secured with crepe bandages pulled under medium to high tension (G–I). During this process, the patient's foot and leg should be relaxed and the ankle held at 90° (J). When the splint has set, the patient is fitted with a Darco® APBTM All Purpose Boot (DARCO (Europe) GmbH), Darco® SlimlineTM Cast Boot (DARCO (Europe) GmbH) or a removable cast walker (K and L). BSD, backslab device.

A step‐by‐step guide to preparation and application of the BSD is shown in Figure 1. Before a BSD is made, the foot wound should be appropriately dressed (Figure 1A). A tubular stockinette is then placed over the foot and leg (Figure 1B). A strip of synthetic splint system/fibreglass (Dynacast® Prelude, BSN Medical) is measured from the patient's plantar forefoot/sulcus to the middle of the calf, taking note of where the heel is located on the splint (Figure 1C,D). The cotton padding encasing the splint is separated to expose the underlying fibreglass sheets (Figure 1E). Edges of the splint should be either trimmed and rounded off or folded down to approximately half an inch within the cotton padding. To ensure conformity to the heel, the edges of the splint (where the heel is) are folded inwards and sprayed with water. The rest of the splint is sprayed with water (on one side) to help it activate and the cotton padding replaced (Figure 1F). This is then placed against the patient's foot and leg, ensuring that any folds are placed away from the skin, and secured with crepe bandages pulled under medium to high tension (Figure 1G–I). During this process, the patient's foot and leg should be relaxed and ankle held at 90° (Figure 1J). When the splint has set, the patient is fitted with a Darco® APBTM All Purpose Boot (DARCO (Europe) GmbH), Darco® SlimlineTM cast boot (DARCO (Europe) GmbH) or a removable cast walker (Figure 1K,L).

The application of a BSD can be done by a single clinician and typically takes approximately 10 min. Post‐application of the BSD, patients are usually instructed to walk a few steps in the clinic room to test for any immediate discomfort or instability. If this occurs, the BSD is discontinued, and the patient reverts to a prior offloading device. An Even‐Up device for the contra‐lateral foot may be used, particularly for patients who use the BSD in a removable cast walker. Patients are given instructions to monitor the BSD closely, and to cease use if any new signs of irritation occurs (usually noted at dressing changes) or if any discomfort or issues with safety is perceived. Follow‐up of the BSD typically involves an in‐clinic review 1 week post‐application, where patients' feet and legs are checked for any new signs of irritation or rubbing, after which the BSD is continually reassessed at each patient's routine Podiatry appointment (as clinically indicated). The BSD is replaced when it starts to lose its rigidity that is, the foot and leg ends can be moved inwards, typically 4–6 weeks post‐application.

### 2.4 Statistical analysis

Descriptive data are presented as percentages, or in the case of continuous variables, as medians with interquartile ranges [IQR].

## 3 DEFINITIONS

Ulcer location was categorised into one of the following: (i) plantar forefoot (to the level of metatarsophalangeal (MTP) joints and not including toes), (ii) plantar midfoot or (iii) plantar heel.

Prior ulcer duration was calculated as the number of days from date of ulcer onset to date of application of the BSD.

Complete ulcer healing was defined by the intact skin at a previous foot ulcer site, meaning complete epithelialisation without any drainage [7] after debridement of callus.

Prior offloading in the 12 weeks before backslab application was categorised into one or a combination of the following (i) TCC, (ii) removable knee‐high device (ProCare® XceltraxTM CAM walker [DJO LLC]), (iii) removable ankle‐high device (Darco® APBTM All Purpose Boot [DARCO (Europe) GmbH], Darco® SlimlineTM cast boot [DARCO (Europe) GmbH] or ProCare® XceltraxTM short CAM Walker [DJO LLC]) or (iv) custom insoles in shoes.

Ulcer area was measured at each patient's routine podiatry and MDFU clinic appointment using the ARANZ SilhouetteStar 3‐D Camera (Aranz Medical) [10, 11, 12].

Vascular status and management of ischemia was recorded as (i) the presence of palpable dorsalis pedis or posterior tibialis pulses on the affected foot, (ii) absolute toe pressures (in mmHg), (iii) history of revascularisation prior to application of the BSD and (iv) revascularisation during application of the BSD.

Wound grading was recorded using the WIfI (Wound, Ischemia and foot Infection) system [13]. Infection management and diabetes care was conducted according to international guidelines [14, 15].

## 4 RESULTS

From the period 1 January–31 December 2021, 28 patients used the BSD as an offloading device in the management of their DFU. One patient had two ulcers on the same foot, each of which were considered a separate ulcer for the analysis. Baseline patient and ulcer characteristics are outlined in Table 1.

**TABLE 1:** Baseline patient and ulcer characteristics prior to BSD application.

Baseline patient characteristics (*N* = 28)			
Demographics	Overall (*N* = 28)	Used BSD (*N* = 19)	Ceased BSD (*N* = 9)
Age (years)	61 (±10)	62 (±8)	57 (±13)
Sex (male/female)	23/5 (82%/18%)	17/3 (61%/11%)	7/2 (25%/7%)
BMI (kg/m^2^)	34 [30–39]	33 [29–39]	34 [32–38]
Diabetes			
Type 1	1 (4%)	1 (4%)	‐
Type 2	27 (96%)	18 (64%)	9 (32%)
Vascular status			
Presence of DP and PT pulses	13 (46%)	9 (32%)	4 (14%)
Presence of one pedal pulse	13 (46%)	8 (28%)	5 (18%)
No pulses	2 (7%)	2 (7%)	‐
Prior revascularisation	5 (18%)	2 (7%)	3 (11%)
Peripheral neuropathy			
Present	28 (100%)	19 (68%)	9 (32%)
Charcot foot deformity			
Present	9 (32%)	8 (28%)	1 (4%)

Baseline ulcer characteristics (*N* = 29)			
Demographics	Overall (*N* = 29)	Used BSD (*N* = 20)	Ceased BSD (*N* = 9)
Site of ulcer			
Plantar heel	9 (31%)	5 (17%)	4 (14%)
Plantar midfoot	7 (24%)	6 (21%)	1 (3%)
Plantar forefoot	13 (45%)	9 (31%)	4 (14%)
WIfI classification: Wound			
Grade 0	‐	‐	‐
Grade 1	17 (59%)	12 (42%)	5 (17%)
Grade 2	12 (41%)	8 (27%)	4 (14%)
Grade 3	‐	‐	‐
WIfI classification: Ischaemia			
Grade 0 (TP ≥ 60 mmHg)	25 (86%)	17 (59%)	8 (27%)
Grade 1 (TP 40–59 mmHg)	2 (7%)	1 (3%)	1 (3%)
Grade 2 (30–39 mmHg)	2 (7%)	2 (7%)	‐
Grade 3 (≤30 mmHg)	‐	‐	‐
WIfI classification: Infection			
Grade 0	28 (97%)	19 (66%)	9 (31%)
Grade 1	1 (3%)	1 (3%)	‐
Grade 2	‐	‐	‐
Grade 3	‐	‐	‐
WIfI amputation risk			
Very low (%)	16 (55%)	11 (38%)	5 (17%)
Low (%)	11 (38%)	7 (24%)	4 (14%)
Moderate (%)	2 (7%)	2 (7%)	‐
High (%)	‐	‐	‐
Prior offloading			
TCC	2 (7%)	1 (3%)	1 (3%)
Knee‐high removable device	6 (21%)	4 (14%)	2 (7%)
Ankle‐high removable device	16 (55%)	11 (38%)	5 (17%)
Custom insoles in shoes	5 (17%)	4 (14%)	1 (3%)

*Note*: Baseline data are summarised as means ± SD, median [interquartile range] or percentages [13].

Abbreviations: BMI, body mass index; BSD, backslab device; DP, dorsalis pedis; PT, posterior tibial; TCC, total contact cast; TP, toe pressure; WIfI, Wound, Ischemia, and foot Infection.

**FIGURE 2:**
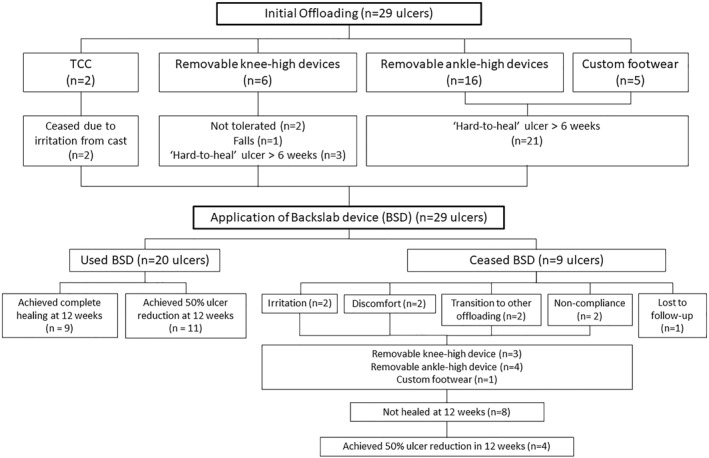
Patient offloading flowchart prior to BSD application and outcomes at 12 weeks post‐BSD application. BSD, backslab device.

Prior to using a BSD, 2 patients (2 ulcers) were offloaded with a TCC but ceased due to issues with irritation. The remaining 26 patients (27 ulcers) were deemed unsuitable for a TCC due to various reasons including: instability, work requirements, obesity, heavily exuding wounds and fluctuant lower limb oedema, and were using other offloading modalities described in Table 1. All patients were recommended to use the BSD as their ulcers continued to persist and were deemed ‘hard‐to‐heal’ despite the use of conventional offloading modalities and good standard care. Out of these, 19 patients (20 ulcers) used the BSD and 9 patients (9 ulcers) ceased BSD use. A diagram outlining prior offloading and rationale for BSD use and respective outcomes is displayed in Figure 2.

**FIGURE 3:**
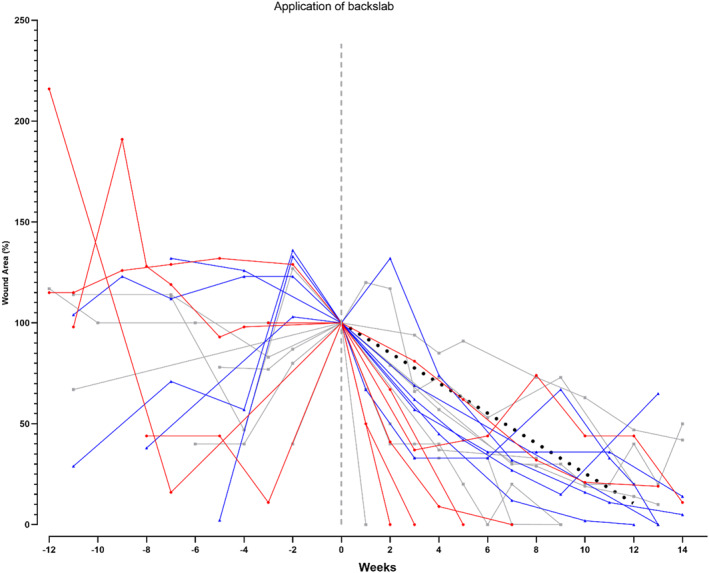
Change in wound area from application of backslab device. Data shown are hindfoot (red), midfoot (blue) and forefoot (grey ulcers). A linear regression line is also shown (black, dashed line).

Prior to initial BSD application, the median (IQR) ulcer area for the 19 patients (20 ulcers) who used the BSD was 1.65 (0.4–3.8) cm^2^ and the median (IQR) duration of the ulcer was 531 (101–635) days. A particular outcome of interest was wound reduction following the first administration of the BSD, measured as a percentage of the baseline wound area (Figure 3). At 12 weeks post‐BSD application, the median (IQR) ulcer area was 0.25 (0–0.55) cm^2^ with a median (IQR) reduction of 97 (80–100) %. Nine (45%) ulcers were found to have achieved complete wound healing in a BSD, with a median (IQR) time to complete healing of 71 (35–134) days. The remaining 11 (55%) ulcers achieved at least 50% reduction in wound size in a BSD, with a median (IQR) time of 24 (15–44) days to achieve 50% reduction in wound size.

**FIGURE 4:**
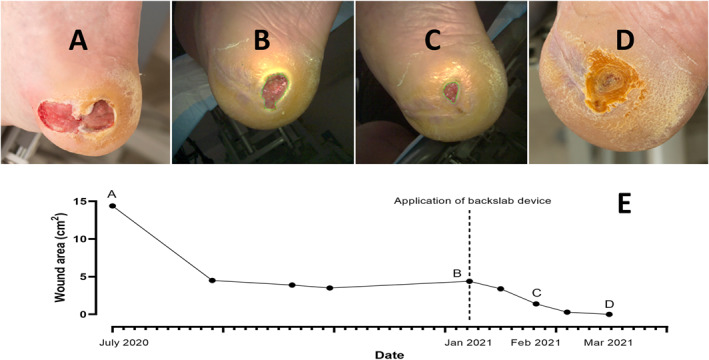
Successful wound healing with a mouldable fibreglass BSD in a patient with a plantar neuropathic heel ulcer (A), in context of Type 2 diabetes mellitus, peripheral neuropathy and morbid obesity (body mass index 41.3 kg/m^2^). This patient was optimised from a diabetes, infection and vascular point of view (palpable pedal pulses and toe pressures of >120 mmHg, not requiring vascular intervention), but effective offloading was a challenge. This patient was unable to tolerate a removable knee‐high walker due to falls, despite the use of a contralateral shoe raise. A TCC or i‐TCC was not feasible due to heavy wound exudate that required daily dressing changes. This patient's only option was to use a pair of custom insoles in removable ankle‐high devices to offload his wound, but this was not successful as the wound remained static for many months. In January 2021, the patient was fitted with a mouldable fibreglass BSD (Dynacast® Prelude, BSN Medical) which was fitted inside a Darco® APBTM All Purpose Boot (DARCO (Europe) GmbH) (B). The patient reported no immediate discomfort or perceived instability when trialled in clinic post‐BSD application. A 50% reduction in wound size was achieved after 3 weeks (C) and complete wound healing occurred in 6 weeks (D and E). Following complete healing the patient was fitted into a WalkOn® AFO (Ottobock SE & Co. KGaA) and a custom insole into off the shelf footwear. This patient has since remained ulcer free. BSD, backslab device; i‐TCC, instant total contact cast; TCC, total contact cast.

Nine (31%) patients ceased use of the BSD due to various reasons, including: transition to other forms of offloading (*n* = 2), irritation (*n* = 2), discomfort (*n* = 2), non‐compliance (*n* = 2) and lost to follow‐up (*n* = 1). Except for one, these patients were documented to have reverted to conventional offloading that had been used prior to application of the BSD (Figure 2). At the 12‐week mark, four of these nine patients had achieved 50% healing in conventional offloading, which included a removable knee‐high device (*n* = 1) and removable ankle‐high devices (*n* = 3), while the remaining four patients experienced no change or an increase in wound size. A comparison of ulcer outcomes in patients who used the BSD and patients that ceased the BSD is summarised in Table 2. An example of successful treatment with the BSD is provided (Figure 4). No BSD complications were documented amongst the patients that used the BSD.

**TABLE 2:** Ulcer outcomes in patients who used BSD and patients that ceased BSD.

Ulcer outcomes	Used BSD (*n* = 20)	Ceased BSD (*n* = 9)
Days ulcer present pre‐BSD	530.5 [98–669]	289 [181–481]
Ulcer size at initial BSD application (cm^2^)	1.65 [0.55–3.8]	2.3 [0.5–4.8]
Ulcer size at 12 weeks post‐BSD (cm^2^)	0.4 [0–0.75]	1.1 [0.15–2.8]^a^
Wound reduction at 12 weeks post‐BSD (%)	92.5 [72–100]	11 [0–79]
Complete ulcer healing at 12 weeks (*n*)	9 (45%)	1 (10%)
50% reduction in ulcer size at 12 weeks (*n*)	11 (55%)	4 (40%)
Time to 50% reduction in ulcer size (days)	27 [16–44]	26 [18–49.5]^b^
Time to complete ulcer healing in BSD (days)	85 [55–273]	‐
Time to complete ulcer healing in standard offloading (days)	‐	671 [206–758]^c^

*Note*: Data are summarised as median [interquartile range] or percentages.

Abbreviation: BSD, backslab device.

^a^ Does not include patient who was lost to follow‐up.

^b^ This value is calculated from the four patients who achieved 50% reduction in ulcer size.

^c^ This value is calculated from three patients who eventually achieved ulcer healing in a removable knee‐high device (*n* = 2) and a removable ankle‐high device (*n* = 1).

## 5 DISCUSSION

The best offloading treatments are those which maximise reductions in high plantar tissue stress without adverse outcomes, whilst maintaining patient satisfaction and, ideally with low cost [1].

While recognised as the gold standard, the TCC has some disadvantages. It is a technically difficult, complex and time‐consuming procedure, requiring skilled expertise by qualified professionals [3, 4, 5, 16, 17, 18]. General guidelines recommend subsequent weekly changes of the TCC [4, 17], but factors such as wound appearance or drainage, wear of the cast, swelling and compliance may require more frequent removal and reapplication [16], which in most cases will not be feasible. Weekly application of a TCC contributes to increased healthcare costs [3, 5, 6, 19], and may also result in patient dissatisfaction [16, 20]. The TCC has been previously shown to be associated with a high risk of treatment‐associated morbidity, including a new cast‐associated blister or wound and amputation following treatment with a TCC, indicating that while the TCC distributes plantar pressure and shear dissipation over a large surface area and has compressive attributes, its rigid nature is not capable of adapting to volume and shape changes, leading to increased shearing forces over bony prominences, resulting in tissue breakdown [21]. The perceived benefit of non‐removability is contra‐indicated in patients with heavily exuding wounds that require frequent dressing changes [18], and also precludes the use of certain wound care products that require frequent changes [21].

While i‐TCC and other removable knee‐high devices may be pragmatic alternatives to TCC they are not always appropriate for patients presenting with morbid obesity, intermittent oedema in the lower limb, and/or severe foot deformity [16, 17]. Offloading options are limited for patients with very large legs (from oedema/obesity) or foot deformities resulting in significant widening of feet, as they are not able to fit into standard removable knee‐high devices. For these patients, removable ankle‐high devices or custom‐made therapeutic footwear are the only options for offloading, but these options may not provide the level of offloading required for wound healing. There are also patients who continue to struggle with ‘hard‐to‐heal’ wounds despite management in a specialist centre according to accepted principles of good standard care, including the use of TCC and removable knee and ankle‐high devices [10].

From this audit, we observed that the BSD was a practical offloading option that resulted in substantial reduction of wound size or complete wound healing in patients who were not able to tolerate the TCC or removable knee‐high walkers, or that had ‘hard‐to‐heal’ wounds despite good standard card and the use of conventional offloading, including TCC. There was, however, a relatively high rate of discontinuation of BSD (31%), consistent with the history of great challenges in achieving effective and tolerable offloading in this patient group.

A benefit of the BSD is that it can be used as an offloading option in patients with foot deformities (resulting in increased width), heavily exuding wounds or fluctuant lower limb oedema because it is made to conform to the plantar and posterior aspects of the foot and leg, instead of circumferentially. In our clinical setting, we have found that it has been successful in providing effective offloading for this particular group of patients who are contra‐indicated for a TCC and do not fit into removable knee‐high devices, who would typically be using a removable ankle‐high devices. Our preliminary findings also suggest that the BSD could potentially be providing more effective offloading when used in a knee‐high or ankle‐high removable device, compared to the use of the device alone, as evidenced by longer ulcer duration prior to the BSD, shorter ulcer duration post‐BSD application and reduction in ulcer area. In theory, the BSD could be safer in patients with mild‐moderate ischemia as the BSD is non‐circumferential and non‐compressive, however clinician discretion would still be strongly advised.

In our experience, a BSD can easily be manufactured within a standard appointment time of 30 min by a single clinician. It is easily removed which allows for close monitoring of infection, as well as frequent dressing changes for patients with highly exuding wounds. Patients are able to remove the BSD for showering and sleeping which adds to overall patient satisfaction and comfort. The light‐weight nature of the BSD compared to TCC or knee‐high removable devices may also contribute towards overall patient acceptability and adherence.

Limitations of the BSD include risks of inadvertent pressure or rubbing to other areas of the foot or leg which may result in a new wound, although this risk is inherent to most offloading devices, including TCC and iTCC. Early follow up and close monitoring of the foot and leg at dressing changes can mitigate this risk.

We acknowledge the limitations with this audit. Being a retrospective observational audit at a single centre, our data may not be generalisable, and subject to biases including observer bias. Next, the number of patients who could safely offload with the BSD was guided by clinical care, which resulted in a small sample size. Lastly, the BSD was not used on patients with ulcers distal to the MTP joints. As such, efficacy of the BSD for patients with hallux plantar interphalangeal joint ulcers or apical ulcers on lesser digits remains unknown. Specific adherence tracking of the BSD device was also not undertaken and is thus unknown.

Despite these limitations, these clinic‐based data support further evaluation in prospective studies. This could include randomised comparative efficacy studies with the TCC or a removable knee‐high walker as comparators, ideally with longer follow‐up duration. A quantitative study involving the measurement of peak plantar pressures pre‐ and post‐application of the BSD compared with TCC and removable knee‐high or ankle‐high devices would also be beneficial, as this may help to guide where the BSD might fit into the current International Working Group on the Diabetic Foot offloading algorithm [6]. There may also be opportunities for research into improved materials to enhance durability of the BSD, as well as opportunities for upskilling of clinicians in the use of BSD, particularly in services where labour and resources may be limited.

## 6 CONCLUSION

Our preliminary findings suggest that the BSD can be used effectively in offloading plantar foot ulcers and may potentially provide better offloading when used in a removable knee‐high or ankle‐high device, compared to use of these devices alone. The advantages of the BSD include: easy application, easy removal which allows close monitoring for infection and frequent dressing changes, light weight and the ability to be used in patients with foot deformities or fluctuant lower limb volume and shape changes. Demonstration of the suitability or efficacy of the BSD as an alternative to conventional approaches will require higher level evidence and further studies are warranted. Nevertheless, BSD presents a practical option for offloading plantar foot ulcers when the TCC or removable knee‐high or ankle‐high offloading devices are contraindicated or not tolerated, or when clinicians are faced with ‘hard‐to‐heal’ wounds despite the use of conventional offloading devices and good standard care [10].

## AUTHOR CONTRIBUTIONS

**Melissa Ting**: Data curation (supporting); formal analysis (supporting); funding acquisition (lead); investigation (equal); project administration (lead); visualization (lead); writing—original draft (lead); writing—review & editing (lead). **Ivana Ferreira**: Data curation (lead); formal analysis (supporting); investigation (equal); writing—review & editing (supporting). **Jonathan Hiew**: Funding acquisition (supporting); investigation (equal); project administration (supporting); resources (lead); writing—review & editing (supporting). **Mahalia McEvoy**: Investigation (equal); writing—review & editing (supporting). **Gabrielle Tan**: Investigation (equal); writing—review & editing (supporting). **Priyal Shah**: Investigation (equal); writing—review & editing (supporting). **Eugenie Nicolandis**: Writing—review & editing (supporting). **Emma J. Hamilton**: Conceptualization (lead); methodology (supporting); formal analysis (supporting); project administration (supporting); supervision (supporting); writing—review & editing (lead). **Jens Carsten Ritter**: Writing—review & editing (supporting). **Michael Nicolaou**: Writing—review & editing (supporting). **Laurens Manning**: Conceptualization (lead); formal analysis (lead); methodology (lead); supervision (lead); validation (lead); visualization (supporting); writing—review & editing (lead).

## CONFLICT OF INTEREST STATEMENT

The authors declare that they have no competing interests.

## ETHICS STATEMENT

Ethical approval was obtained from the South Metropolitan Health Human Research Ethics Committee (RGS0000003204).

## CONSENT FOR PUBLICATION

Written, informed consent for publication of patient images was provided by the patient.

## Data Availability

All relevant data is contained within the article.
